# Personalized Text Messages and Automated Calls for Improving Vaccine Coverage Among Children in Pakistan: Protocol for a Community-Based Cluster Randomized Clinical Trial

**DOI:** 10.2196/12851

**Published:** 2019-05-30

**Authors:** Abdul Momin Kazi, Nazia Ahsan, Ayub Khan, Saima Jamal, Hussain Kalimuddin, Naveera Ghulamhussain, Zabin Wajidali, Abdul Muqeet, Fabiha Zaidi, Meraj Subzlani, William McKellin, Asad Ali, Jean-Paul Collet

**Affiliations:** 1 Department of Pediatrics and Child Health Aga Khan University Karachi Pakistan; 2 Department of Pediatrics University of British Columbia and BC Children’s Hospital Research Institute Vancouver, BC Canada; 3 Digital Health Resource Center Aga Khan Development Network Karachi Pakistan; 4 Department of Sociology University of British Columbia Vancouver, BC Canada; 5 Beijing Tiantan Hospital and Advanced Innovation Center for Human Brain Protection Capital Medical University Beijing China

**Keywords:** routine immunization, SMS messages, automated call messages, cluster randomized clinical trial, vaccine barriers, personalized intervention, cell phones, vaccination coverage, mobile health, text messaging, developing countries, parents

## Abstract

**Background:**

A major reason for poor childhood vaccine coverage in developing countries is the lack of awareness among parents and caregivers regarding the need for immunization and the importance of completing the entire series of vaccines. Short message service (SMS)–based interventions have been quite effective in different programs such as smoking cessation, treatment adherence, health care scheduled appointment attendance, antenatal care attendance, and compliance to immunization. However, there are limited data from low- and middle-income countries on the role of SMS and automated call–based messages and interventions to improve routine immunization (RI) coverage.

**Objective:**

The primary objective of this study is to evaluate whether automated mobile phone–based personalized messages (SMS or automated call) can improve RI uptake at 6, 10, and 14 weeks of age per the expanded program immunization schedule, compared with a usual care control group. Secondary objectives include assessing the effects of different types of automated SMS text or calls on RI coverage at 20 weeks of age.

**Methods:**

This is a mixed methods study using a clustered randomized controlled trial with 4 intervention arms and 1 control arm, augmented by qualitative interviews for personalizing the message. The study is being conducted in Pakistan (an urban site in Karachi and a rural site Matiari). In Karachi, 250 administrative structures are taken as 1 cluster, whereas in Matiari, a catchment area of 4 Lady Health Workers is considered as 1 cluster. The intervention targets families to receive weekly 1-way or 2-way (interactive) personalized automated SMS or automated phone call messages regarding vaccination. Possible barriers to vaccination are assessed in each family at the time of inclusion to determine the type of personalized messages that should be sent to the family to increase the chance of a positive response. Finally, in-depth interviews using purposive sampling are conducted before and after the trial to determine the family’s vaccination experience and related factors.

**Results:**

All study participants for the cluster randomized trial were enrolled by January 14, 2019. Study exit interviews at 20-weeks follow-up visits will be completed by June 2019.

**Conclusions:**

The results of this study will be useful to understand the respective effects of SMS text messages versus automated phone–based communication to improve RI coverage and timelines. Moreover, information regarding families’ perceptions of vaccination and the daily life challenges for timely visits to the vaccine clinic will be used for developing more complex interventions that use mobile phone messages and possibly other approaches to overcome barriers in the uptake of correct and timely immunization practices.

**Trial Registration:**

ClinicalTrials.gov NCT03341195; https://clinicaltrials.gov/ct2/show/NCT03341195 (Archived by WebCite at http://www.webcitation.org/78EWA56Uo)

**International Registered Report Identifier (IRRID):**

DERR1-10.2196/12851

## Introduction

### Background

Pakistan is one of the countries with the highest rates of child death in the world [1]. It ranks 4th in child mortality, with 60% deaths due to vaccine-preventable diseases (VPDs) [2]. Table 1 [18] shows the current schedule of routine immunization (RI) in Pakistan, which is provided by the government free of cost. The immunization coverage in Pakistan is estimated to be 59%, which is still well below the desired level, leading to continued polio transmission, large measles outbreaks, and thousands of deaths from vaccine-preventable illnesses [2]. In addition, Pakistan is a major polio epidemic country and among 3 countries in the world requiring proof of polio vaccination for international travel [3]. Pakistan demographic and health survey in 2017-2018 suggests 88% percent of children had received BCG vaccine due at birth, 86% and 95% had received the first dose of pentavalent and polio vaccine respectively due at the 6^th^ week. Furthermore, 75% and 86% of children had received the third dose of the pentavalent and polio vaccines, respectively, due at 14^th^ week and measles vaccination was 73%, which is due at 9 months. However, these rates are at 1 year of age and much higher than vaccination coverage rate at scheduled time and among conflict hits and displaced populations [4]. Improved RI coverage is recommended as the priority public health strategy to reduce VPDs and eradicate polio in Pakistan and worldwide.

According to immunization coverage surveys, 1 in 5 children is unimmunized [5]. A major reason for poor childhood vaccine coverage is low immunization uptake, when parents are unable to complete the entire series of vaccines in accordance with the scheduled timelines. Some of the reasons include: (1) the family is not in favor of getting their child immunized, (2) low trust in vaccines provided through Expanded Programme on Immunization (EPI) and government health care providers, and (3) caregivers have forgotten their child’s next vaccination due date or child’s EPI card is misplaced [6]. These barriers may be modified with additional support through education and behavior change strategies. In addition, with more pressing issues of food and shelter, preventive health often takes the back seat, and parents and caregivers forget or ignore the subsequent doses of vaccines for their children. There is an immense need to encourage parents’ care seeking and collaboration with the health care providers to improve initial vaccine uptake and the completion of all doses according to the schedule. New innovative and cost-effective techniques are necessary for practical solutions to improve vaccination uptake and coverage.

Mobile phones offer a new medium to provide education and advocate families or caregivers to enable behavior change so as to improve immunization uptake. Mobile phone use has also increased in countries with low RI coverage and a high risk of VPDs. Good examples are Nigeria and Pakistan, where there were around 170 and 140 million mobile phone subscribers, respectively, in 2014 [7,8]. There is also a surge in use of short message service (SMS), with 237.58 billion person-to-person SMS generated in 2011 estimating to around 175 SMS per mobile phone on a monthly basis in Pakistan [9].

**Table 1:** Childhood immunization schedule for Pakistan.

Disease	Causative agent	Vaccine	Doses	Age of administration
Childhood tuberculosis	Bacteria	Bacillus Calmette-Guerin	1	Soon after birth
Poliomyelitis	Virus	OPV (oral poliovirus vaccine); Inactivated polio vaccine (IPV)	4; 1	OPV0: soon after birth, OPV1: 6 weeks, OPV2: 10 weeks, OPV3: 14 weeks; IPV-1: 14 weeks
Diphtheria, Tetanus, Pertussis, Hepatitis B, Hib pneumonia, and meningitis	Bacteria	Pentavalent vaccine (Diphtheria, tetanus toxoids and pertussis +Hepatitis B + Hib)	3	Penta1: 6 weeks, Penta2: 10 weeks, Penta3: 14 weeks
Pneumonia	Bacteria	Pneumococcal vaccine (PCV)	3	PCVIO 1: 6 weeks, PCVIO 2: 10 weeks, PCVIO 3: 14 weeks
Measles	Virus	Measles	2	Measles1: 9 months, Measles2: 15 months
Diarrhea due to rotavirus	Virus	Rotavirus	2	Rota 1: 6 weeks, Rota 2: 10 weeks

Mobile reminders in the form of phone calls have also proven to be a feasible method of improving RI uptake in resource-limited settings with wide cellphone coverage [10]. A Cochrane review reported that automated telephone communication systems including automated calls are effective in a variety of health care settings such as improving clinic attendance rates, screening, adherence to medications, and laboratory tests [11]. Automated calls were also found to be cost-effective in increasing immunization rates in an urban practice in the United States [12]. A study suggests that mobile phones have wide spread abilities to improve health outcomes in low and middle-income countries (LMICs) by targeting larger populations in a cost-effective manner [13]. Furthermore, SMS-based reminders along with small financial incentives could possibly help in improving RI timelines [14]. Text reminders have also proved to reduce vaccine dropout rate and improve parents’ compliance to immunization-scheduled visits [15,16].

There are limited data from LMICs set up on the role of SMS-based interventions for improvement of RI coverage, and conventional 1-way reminder SMS text messages were used by most of the studies as the intervention [13,21,22]. Overall, very few studies compared reminders, educational, and interactive SMS messages related to childhood vaccination uptake [13,21-26]. Although some of the studies have shown some behavior change for improvement in vaccination coverage, more rigorous application of health behavior change model needs to be applied to understand the impact of reminder, educational, and interactive messages on behavior change related to improvement in RI coverage [17]. However, data from developing countries regarding the role of automated calls in improving vaccine coverage are limited. Majority of the studies have focused on SMS-based intervention.

In this study, we will identify the factors that affect adherence to RI coverage among Pakistani families and caregivers and will examine an important public health question—do low cost, automated SMS text messages and calls improve RI coverage in resource-constrained settings such as Pakistan? In this study, we will compare the effectiveness of different types of messages: reminder, educational, and interactive SMS text messages and automated calls for improving RI uptake. Different types of messages will be developed to meet the possible RI barriers identified; these messages will be sent specifically to the participants according to the type of RI barriers they faced for immunization. This information will be used to develop strategies to improve vaccine adherence in Pakistan.

### Study Objectives

#### Primary

Our first objective is to evaluate whether personalized automated SMS text messages, 1-way versus 2-way, can improve on-time visits at 6, 10, and 14 weeks of age for RI as compared with standard care. Our second objective is to evaluate whether personalized automated calls, 1-way versus 2-way, can improve on-time visits at 6, 10, and 14 weeks of age for RI as compared with standard care. Finally, our third objective is to compare the efficacy of automated SMS text messages versus automated calls on increasing vaccination uptake.

#### Secondary

Our secondary objectives are to learn the perception and attitudes of caregivers regarding childhood vaccination and to find out about factors that might influence mobile phone and SMS text–based interventions for vaccination improvement.

## Methods

### Target Population

Our target population are caregivers of newborn (NB) infants younger than 14 days who are due for their RI at age 6, 10, and 14 weeks according to the EPI schedule of Pakistan.

### Study Goal

Our goal is to identify the barriers faced by families in the uptake of correct immunization practices and to develop adapted messages (SMS or automated calls) to improve vaccine adherence in Pakistan. We will collect information from families regarding perceived sociocultural, technical, and economical barriers that may explain the decrease in vaccine coverage, and possible solutions to overcome these constraints. This information will be used to develop personalized educational messages, reinforced by interactive exchanges (2-way automated SMS and automated calls) with caregivers in the initial 20 weeks of their child’s life.

### Study Hypothesis

Personalized weekly message in the form of automated SMS text message or automated calls according to barriers for RI and language preferences can improve RI uptake according to the schedule for vaccine due at age 6, 10, and 14 weeks as compared with standard counseling by health care providers at EPI center and outreach visits for RI. Interactive voice recording (IVR) or 2-way auto calls might be preferred way of communication to caregivers as compared with other intervention and control for possible improvement in RI coverage and timelines.

### Outcomes

#### Primary

Primary outcomes include: (1) to see a 10% increase in RI through personalized automated mobile phone–based communication (SMS or automated call) at 6, 10, and 14 weeks of age according to the EPI schedule versus standard care; (2) to see a 10% increase in RI within 1 week of the original timeline at 6, 10, and 14 weeks versus standard care; (3) to compare coverage rates of personalized 1-way versus 2-way SMS text messages on improvement in RI at 20 weeks of age; (4) to compare coverage rates of personalized 1-way versus 2-way automated calls on improvement in RI at 20 weeks of age; and (5) to compare coverage rates of personalized SMS text messages and automated calls on improvement in RI at 20 weeks of age. 

#### Secondary

Secondary outcomes include: (1) to understand the perceptions and barriers of caregivers regarding immunization and (2) to understand the perception of caregivers related to personalized mobile phone–based SMS text messages and automated health messages for vaccination improvement.

### Methodology

#### Study Design

**Figure 1:**
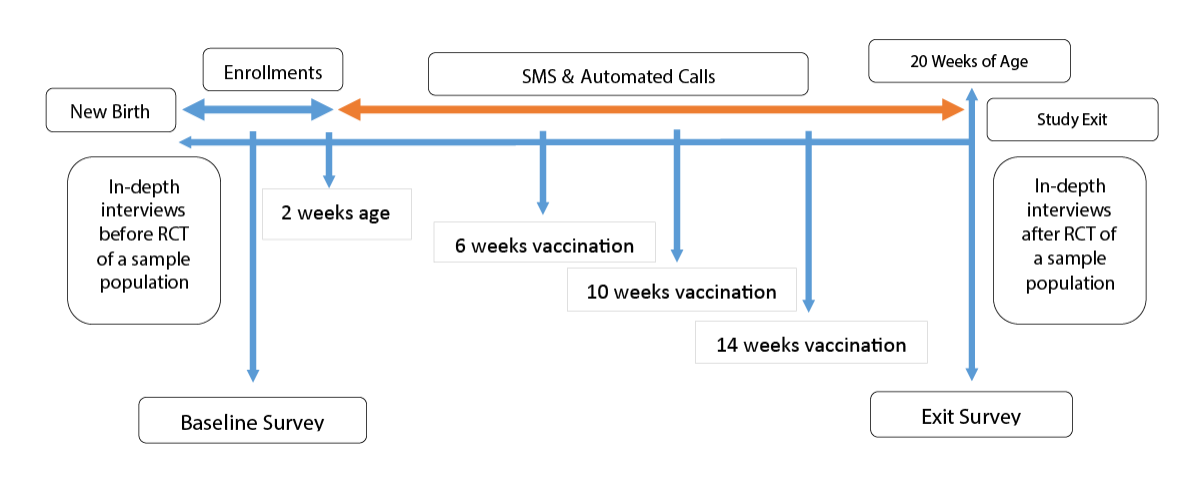
Study design: a mixed methods randomized controlled trial (RCT) augmented by qualitative interviews. SMS: short message service.

The study design is a mixed method clustered randomized controlled trial (RCT) augmented by qualitative interviews (Figure 1). The cluster trial will be used to assess the respective impacts of mobile phone and SMS-based communications on RI coverage rates among children at 20 weeks of age. Initially, structured interviews will be conducted at baseline to identify barriers to immunization and role of SMS and automated call–based messages in improving vaccination coverage. This information will lead to the development of personalized barrier-specific messages (SMS and voice calls) that will be used for each participant or caregiver according to the specific barriers they struggle with, as identified at baseline.

#### Study Sites

**Figure 2:**
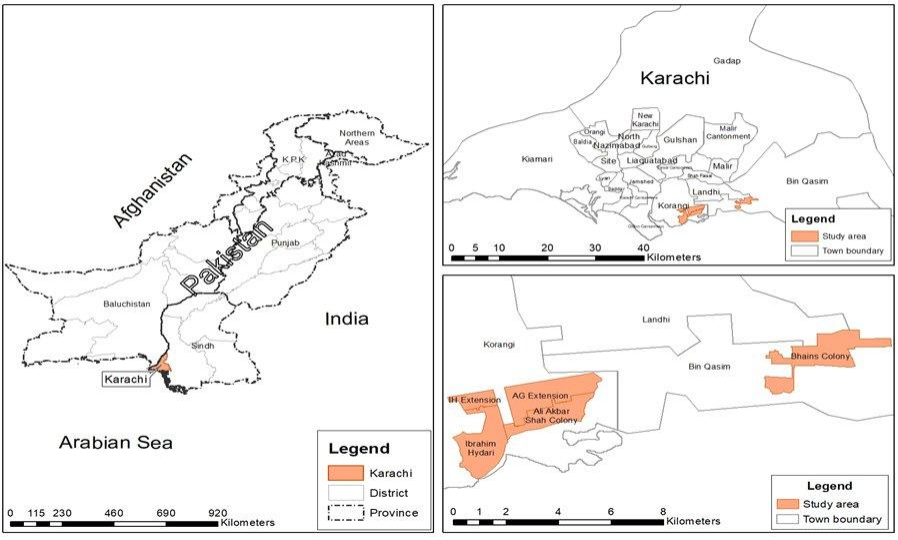
Catchment area of Karachi pre-urban demographic surveillance site.

The Department of Paediatrics and Child Health at the Aga Khan University (AKU) conducts active health demographic surveillance systems (HDSS) at several urban sites in Karachi. These sites are (1) Ibrahim Hydri Goth, (2) Ali Akber Shah Goth, (3) Rehri Goth, and (4) Bhains colony, which is part of Bin Qasim Town (Figure 2). Ibrahim Haidry Goth, Ali Akber Shah Goth, and Rehri Goth are located along the sea coast of Karachi, and the main occupation of people living in these communities is fishing. Bhains Colony is located at the outskirts of Karachi, and the main source of income of its population is dairy products.

The HDSS sites are divided into 195 blocks, each containing 250 structures and mapped using the geographical information system. There are a total of 42,093 structures where 43,098 households are living in areas of Korangi and Bin Qasim towns [19]. A *structure* is defined as a building with a single entrance and a boundary. These structures can be houses, hospitals, dispensaries, schools, shops, parks, etc. Each structure has a unique number assigned to it. The total population of the active surveillance catchment area is approximately 0.3 million, with around 7115 pregnant women and 6831 NBs being followed annually by the surveillance team. Within 1 block, the 250 administrative structures will be our trial clusters; 4 sites will be participating in the study. NBs will be enrolled from the HDSS, and clusters within the 4 sites will be randomly assigned to the study arms. Hence, participants within each administrative structure (ie, cluster) will receive same intervention.

**Figure 3:**
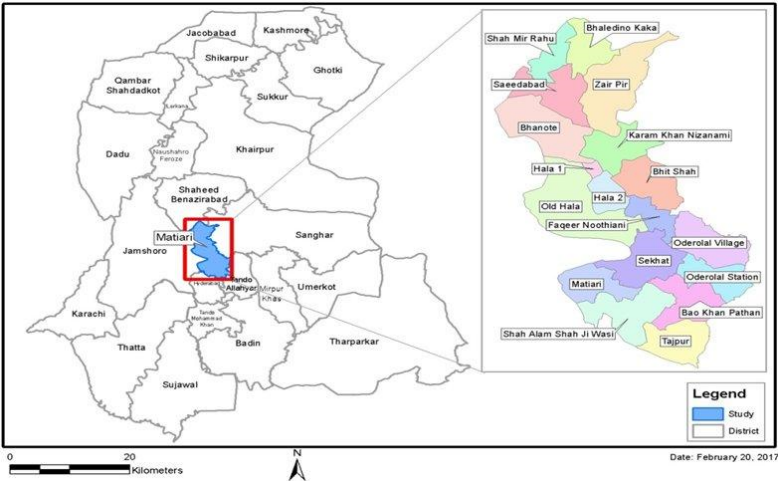
The Matiari site: catchment area of surveillance site.

Data will also be collected from the rural site of Matiari, which is located in Sindh province 185 km north of Karachi (Figure 3). The department of pediatrics will partner with the local Lady Health Worker (LHW) program Sindh. Information about new births within LHW catchment area will be provided to the study team. The catchment areas in Matiari are the areas covered by each LHW; these areas are small and that is why the catchment area of 4 LHWs will be considered as 1 cluster. The total population of the Matiari District area is approximately 0.4 million. A total of 3 main sites from district Matiari will be the part of study, which includes Matiari, Hala, and Saeedabad. The main source of income in this area is agriculture. In Matiari and Karachi, clusters will have a mean of 15 births per cluster.

#### Baseline Survey

A total of 50 interviews will be conducted from Karachi and Matiari sites (ie, 25 interviews from each site). From the HDSS, list of caregivers who have completed the vaccination schedule and those who are dropouts or not vaccinating their child was taken out, and randomly, caregivers will be selected for in-depth interviews. Trained staff will conduct the qualitative interviews. Depending upon the availability, caregivers will be approached at the household, and appointments will be taken from the caregiver so that they can spare enough time for the discussion. Through in-depth interviews, we will explore the perceptions and barriers related to EPI and mobile health from the caregivers. Information collected before the study through qualitative in-depth interviews will help in developing the survey to assess the different categories of barriers. The baseline survey will, overall, collect information on demographics, mobile phone access and usage, possible barriers for RIs, and factors associated with mobile phone–based health messages.

#### Qualitative Phase

##### Assessing Perceptions and Experiences of Caregivers About the Routine Immunization and the Role of Mobile Phone Short Message Service and Automated Calls in Improving the Routine Immunization Coverage and to Develop Messages

Initially, in-depth interviews with a subpopulation in the study catchment area will be conducted to explore RI immunization coverage among Pakistani caregivers and families to assess (1) perceptions regarding risks of infectious diseases preventable by vaccines and vaccine safety and efficacy, (2) barriers to vaccinating children including difficulties in visiting RI centers, and (3) perceptions and barriers that may affect mobile phone–based interventions to improve immunization coverage. The interviews will be conducted across the site regions. We will use purposive sampling to assure that participants represent different ethnic groups. Information gathered through the interviews will help in (1) understanding the types of barriers perceived by caregivers, (2) designing the RCT and, (3) developing content for SMS text messages in several categories of barriers. We expect 20 to 25 parents or caregivers to be interviewed over a period of 3 months, but the number will be guided by saturation of information. Each interview will take 45 to 60 min.

### Randomized Clinical Trial

The study design is a cluster randomized clinical trial including 4 intervention and 1 control arm (Table 2).

#### Sample Size Calculations

Assumptions used for calculating sample size are as follows: increase in coverage rate from 30% to 40%, having a power of 80% with an alpha error at .05. The sample size per arm would be 615 per arm or 3075 for all 5 arms. Adding a 10% dropout will make it 677 per arm or 3385 recruitment for all 5 study arms. The ICC is taken as .05, with an estimate of 15 NB per cluster (see Table 3).

**Table 2:** Cluster randomized clinical trial.

Study arm	Automated SMS^a^ text and phone calls			
	1-way SMS text	2-way SMS text	1-way phone calls	2-way phone calls
1	X^b^	—^c^	—	—
2	—	—	X	—
3	—	X	—	—
4	—	—	—	X
5	—	—	—	—

^a^SMS: short message service.

^b^Applicable.

^c^Not applicable.

**Table 3:** Sample size calculation. Having a difference of 10% and the sample size given (ie, 1230 is 2 groups, 1:1) in our study, we have 5 groups (615x5)+10% dropout=3385.

Serial #	Interclass correlation	Range of cluster size					
		Cluster size of 15		Cluster size of 20		Cluster size of 30	
		Clusters, n	Total birth required, n	Clusters, n	Total birth required, n	Clusters, n	Total birth required, n
1	0.01	54	810	44	880	32	960
2	0.05	82	1230	70	1400	58	1740
3	0.09	108	1620	98	1960	86	2580
4	0.1	114	1710	104	2080	94	2820
5	0.13	134	2010	124	2480	114	3420
6	0.15	148	2220	138	2760	128	3840
7	0.2	180	2700	172	3440	162	4860
8	0.21	188	2820	178	3560	168	5040
9	0.25	214	3210	206	4120	196	5880
10	0.3	248	3720	238	4760	230	6900
11	0.35	280	4200	272	5440	264	7920
12	0.37	294	4410	286	5720	278	8340
13	0.4	314	4710	306	6120	300	9000
14	0.45	346	5190	340	6800	334	10,020
15	0.5	380	5700	374	7480	368	11,040

### Enrollment Criteria

#### Inclusion Criteria

The inclusion criteria include being a child from the HDSS site, being younger than 14 days with a parent or guardian or at least 1 person in the household, having a working mobile phone connection, and parent or guardian providing consent to participate in the study.

#### Exclusion Criteria

The exclusion criteria include a child from outside the HDSS area or if the family plans to stay in the catchment area for less than 20 weeks.

### Sampling Strategy and Randomization

**Figure 4:**
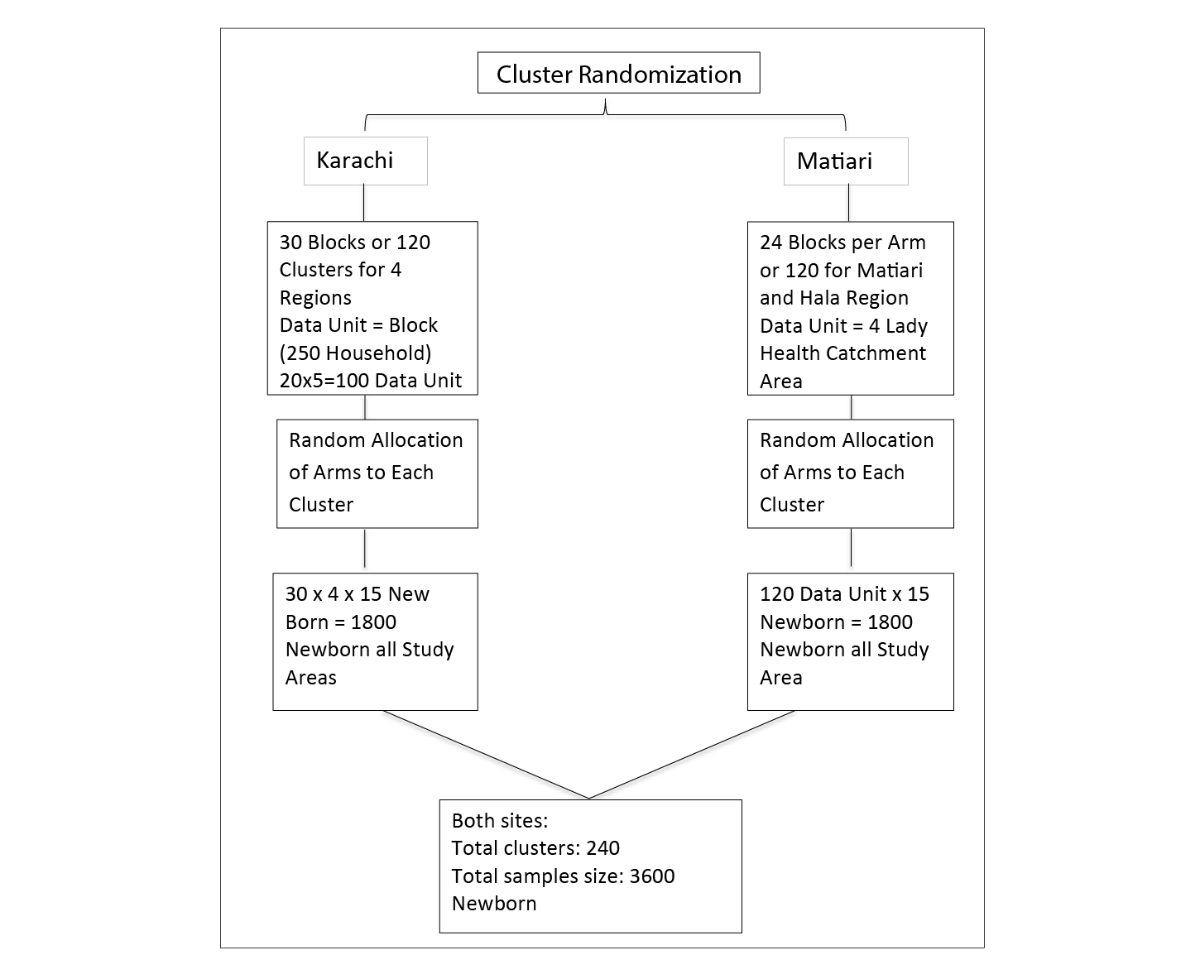
Cluster Randomization.

The sampling strategy adapted for both sites are as follows (Figure 4).

### Karachi Site

The data for live births and population of each site in Karachi will be obtained from HDSS team. Data will be scrutinized and divided according to 4 sites in the study, with each site consisting of 5 arms (5 clusters). Administrative structures will be assigned to each intervention through stratified (ie, site) randomization.

### Matiari Site

**Figure 5:**
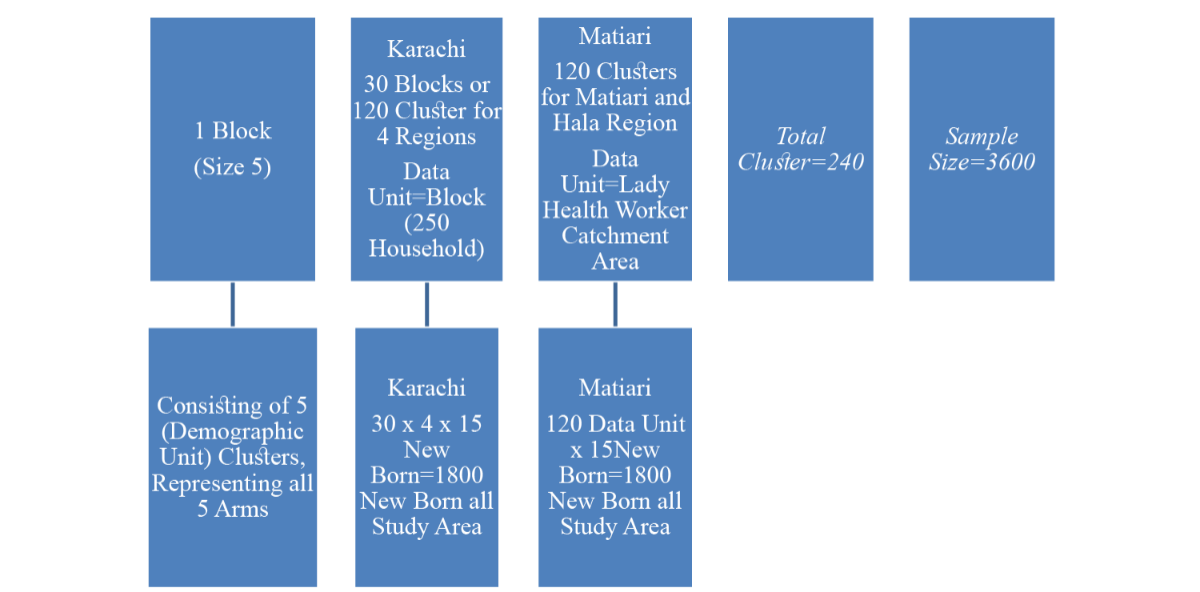
Sampling strategy for Karachi and Matiari. For 10% effect size, sampling strategy cluster size=15.

As Matiari site has not been stratified into clusters according to HDSS, we randomly allocated 30 clusters per arm. The catchment area of 4 LHWs will be considered as 1 cluster. Each intervention arm will then be randomly allocated to 24 administrative structures (clusters; see Figure 5).

### Recruitment

For Karachi, birth data of the clusters will be shared by central HDSS team on weekly basis and at Matiari the LHWs will inform births to the study team daily according to the study clusters. The study staff will approach families who have an infant younger than 2 weeks in each cluster.

Staff will explain the study objectives to the parents or caregivers. If the parent or caretaker is interested in the study, the infant will be evaluated for enrollment. One child per household will be selected. In a household, where there is more than 1 child (younger than 14 days), a random selection will be made by a program designed in the mobile phone device. After meeting the eligibility criteria, informed consent will be obtained, and the infant will be enrolled in the study and a baseline survey will be conducted.

### Follow-Up Process

**Figure 6:**
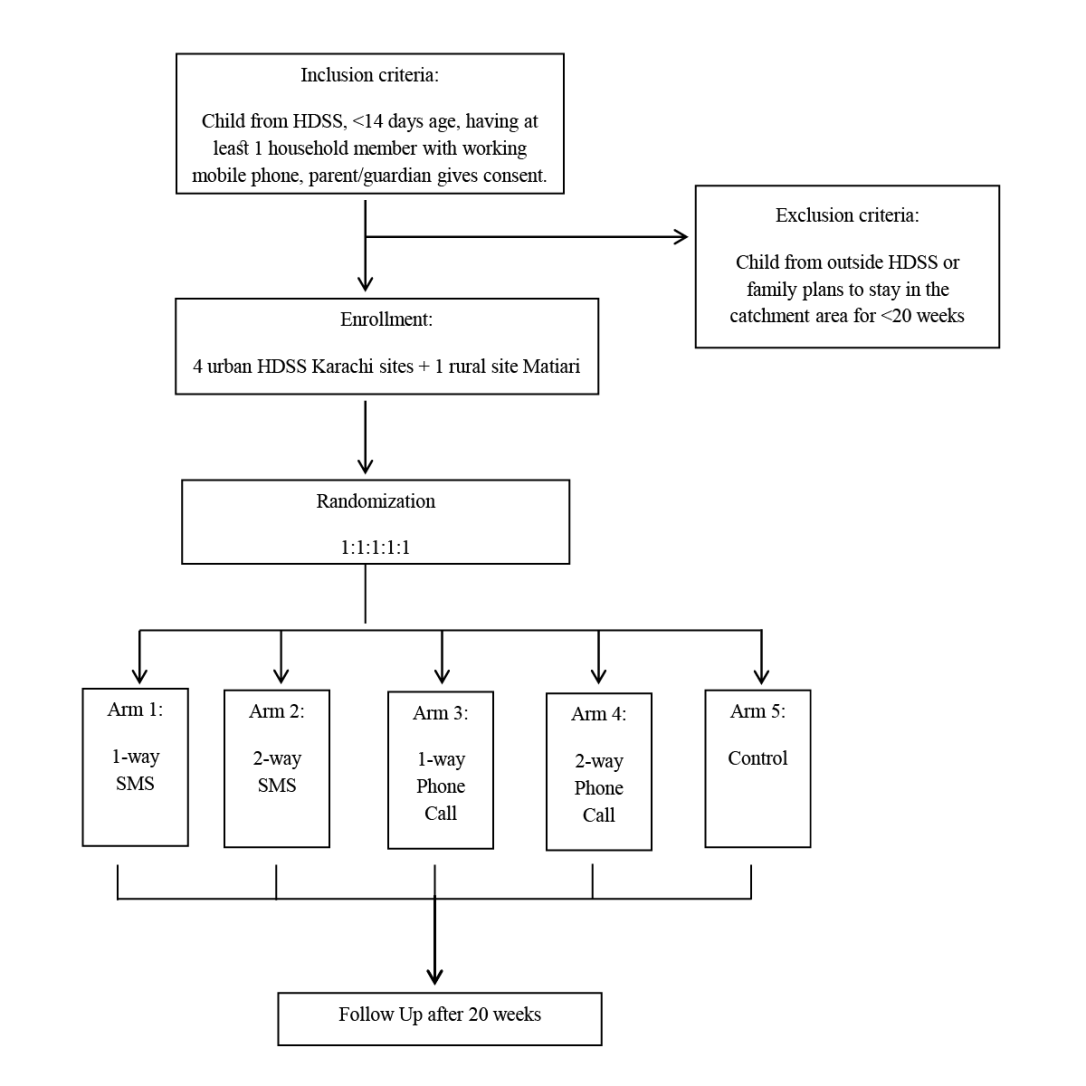
Participant flow diagram. HDSS: health demographic surveillance systems; SMS: short message service.

A second survey will be conducted at 20 weeks of child’s age (end of follow-up) to identify vaccination coverage according to the schedule, to be confirmed by physical examination of EPI card (see Figure 6).

### Study Intervention

#### Intervention Group

**Figure 7:**
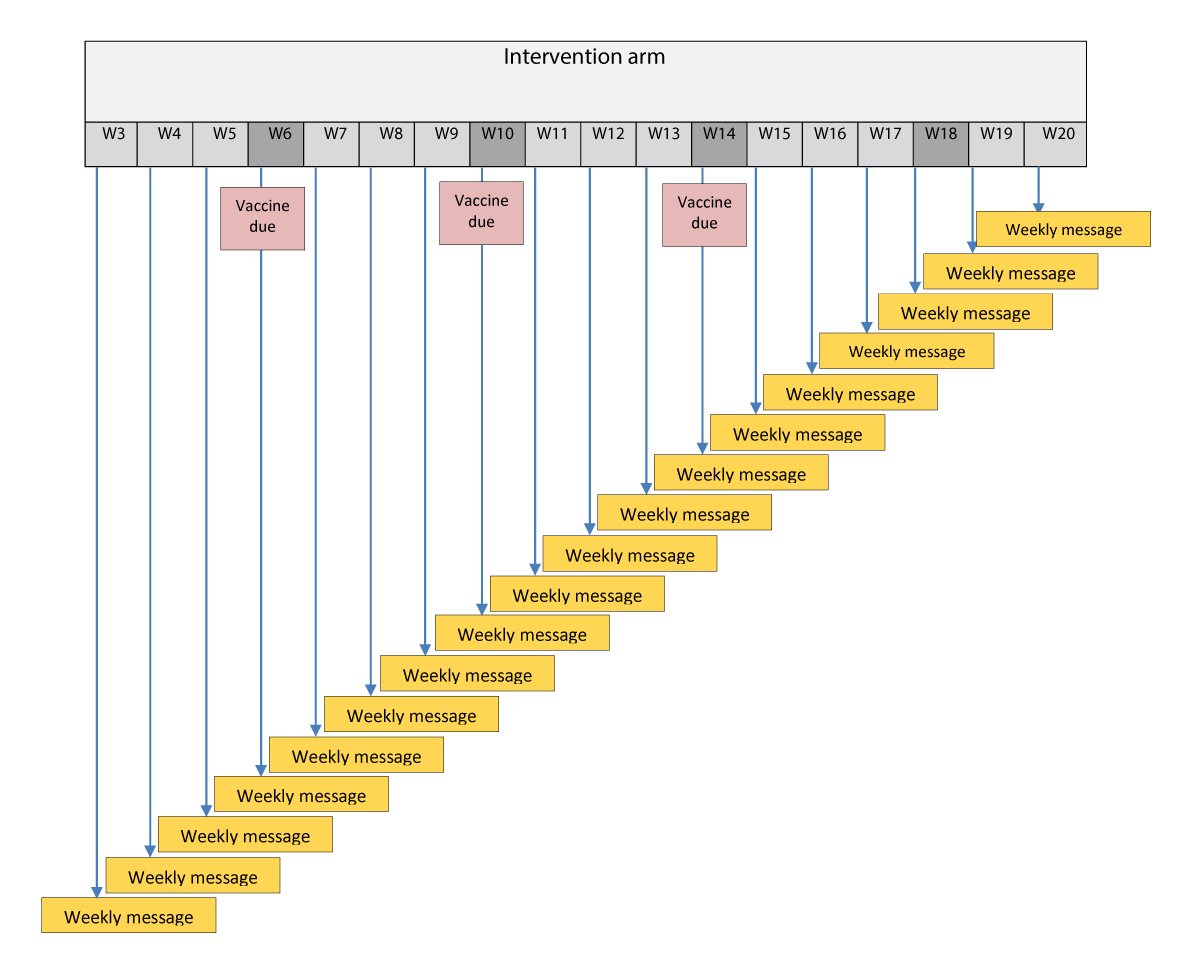
Details of study arm explained.

The intervention arms in addition to the standard counseling will include receiving personalized 1-way or 2- way (interactive) automated SMS or 1-way or 2-way (IVR) automated phone call messages regarding vaccination. In personalized 1-way messages and automated calls, parents will receive educational or reminder or proactive messages related to RI once a week till the child turns 20 weeks. SMS and automated phone calls will contain the same information content and interactive features, only the delivery method will differ. In the interactive arms, in addition to personalized weekly educational or reminder or proactive message, parents will have the option to reply to messages or calls and receive more information related to immunization through SMS text messages or calls (see Figure 7 and Table 4 and Multimedia Appendices 1 and 2). The study timeline has been provided in Table 5.

#### Control Group

The control group will receive 1 time standard verbal counseling at the time of initial visit for on-time EPI vaccines at 6, 10, and 14 weeks of age as recommended by EPI, government of Pakistan.

**Table 4:** Detail on the personalized weekly short message service and automated calls from enrollment to 20 weeks of life.

Intervention arm	Weekly automated SMS^a^ text message and automated calls from enrollment till 20 weeks of life
Arm 1 (intervention)	Parents or caregivers will receive 1-way educational or reminder or proactive SMS messages related to routine immunization once a week till 20 weeks of age
Arm 2 (intervention)	Parents or caregivers will receive 2-way (interactive) educational or reminder or proactive SMS messages related to routine immunization once a week till 20 weeks of age—parents will have the option to reply and receive more information related to immunization through SMS text messages
Arm 3 (intervention)	Parents or caregivers will receive 1-way educational or reminder or proactive automated phone call related to routine immunization once a week till 20 weeks of age
Arm 4 (intervention)	Parents or caregivers will receive 2-way (interactive) educational or reminder or proactive automated phone call related to routine immunization once a week till 20 weeks of age—parents will have the option to reply and receive more information related to immunization through phone call
Arm 5 (control)	One time counseling at the baseline survey

^a^SMS: short message service.

**Table 5:** Study timeline.

Steps	2018						2019			
	Jan/Feb	Mar/Apr	May/Jun	Jul/Aug	Sep/Oct	Nov/Dec	Jan/Feb	Mar/Apr	May/Jun	Jul
Protocol development	X^a^	—^b^	—	—	—	—	—	—	—	—
Ethical review submission	X	—	—	—	—	—	—	—	—	—
Standard operating procedures	X	—	—	—	—	—	—	—	—	—
Training and implementation of field team	X	—	—	—	—	—	—	—	—	—
Qualitative interviews	X	X	X	—	—	—	—	—	—	X
Trial recruitment	—	—	—	X	X	X	X	—	—	—
Trial follow-up	—	—	—	—	—	X	X	X	X	—
Data entry cleaning and analysis	—	—	—	X	X	X	X	X	X	—
Report and scientific paper writing	—	—	—	—	—	—	—	—	X	X
Thesis completion	—	—	—	—	—	—	—	—	X	X
Additional—baseline, analyses	—	—	—	—	—	—	—	—	X	X

^a^Applicable.

^b^Not applicable.

### Study End Interview

Interviews will be conducted after the trial, with a purposive sample of a subpopulation included in the RCT representing all study arms. From these structured in-depth interviews, we expect to further explore participants’ experience of the different study arms having different levels of coverage. We will also assess the factors related to SMS text messages and automated calls associated with vaccination uptake. Sample size will be guided by saturation of the information.

### Statistical Analysis

Analyses will be conducted according to the intention-to-treat principle. When the information cannot be gathered, the vaccine status of the child will be *failure* (conservative approach). Missing the vaccine EPI card to verify vaccine status at home will lead to a deeper investigation in the family and also the vaccine clinic. From previous experience, we expect this process to be successful in identifying the outcome of over 95% (95/100) of the children who remain in the study at 20 weeks. Families that have left the study for any reason (moving, withdrawal, child death) will be interviewed (if possible) and the information gathered used for the outcome classification. If the information is not clear or incomplete, the child’s status regarding vaccination will be *failure*.

The main analyses will be comparing our 4 study intervention arms against the control, and with each other. For the purposes of our study, we will compare each of the 4 interventions with each other and with the control arm. For example, 1-way versus 2 SMS, reminder versus educational SMS, 1-way reminders SMS versus control, 1-way educational SMS versus control, 2-way reminders SMS versus control, 2-way educational SMS versus control, 2-way education versus 1-way reminder, and 2-way reminder versus 1-way educational.

### Analysis of Quantitative Data

Analyses will be conducted according to the intention-to-treat principle, and the Bonferroni correction will be used to control for multiple testing. The primary outcome is to assess difference in vaccination status. Chi-square test will first be used to compare the groups’ percentage. Multivariable logistic regression analyses will be conducted to adjust for confounders. As our randomization unit is cluster-based rather than individual-based, generalized linear mixed effects models or generalized estimating equations models will be employed to account for within-cluster correlation, and hierarchal models will be used. The statistical model for the primary and secondary analyses will be developed as explanatory variables. As secondary analyses of the primary outcome, time-to-immunization curves will be constructed using the Kaplan-Meier method and Cox regression for multivariate analysis.

### Analyses of Qualitative Component

In the first section of the semistructured interview guide, we will ask the caregivers regarding common barriers they encounter at the time of RI, whereas the second section comprises perceptions and barriers related to a mobile phone. The data will be transcribed into written form from audio recordings and will be analyzed via qualitative data analysis software NVivo 11 (QSR International). Written transcripts will then be uploaded into NVivo 11 software to offer easy and organized retrieval of data for analysis. The data analysis will be conducted according to discourse analysis. The interview guides will also be pretested in a similar community

### Data Management, Confidentiality and Privacy Protection, and Quality Assurance

The audio recording and transcripts will have a unique identifier; original and backup files will be archived in password-protected computer or server at AKU. Only transcriptions and themes will be shared at University of British Columbia (UBC) without nominative information. All study-related data including the recording will be stored in an encrypted server with password protection and having access only to the study specific personnels.

Baseline and end line data will be collected on a smartphone device (Multimedia Appendices 3 and 5). The entry program will be designed to capture data as well as the location of the interviewer along with some monitoring parameters. Each child participant will be given a structured unique identifier. Business rules, skips, and consistency checks will be incorporated, and important fields will be marked as must enter in the questionnaire to maintain data collection quality. The database will reside on a central computer at AKU managed by the study staff. A Web-based dashboard will be designed to report daily study progress. Mobile numbers will not be shared except to track patterns of use. Only relevant study staff will have access to study data allowed by the local ethics committee. All study staff will undergo basic research ethics training. Participants’ information will be given a study code, and no personal identifiers will be shared. Data confidentiality will be maintained at all times. No personal identifiers will be used in any reports or publication of the study. Mobile phone companies will be only provided mobile phone numbers to send SMS text messages and automated calls messages through gateway (Multimedia Appendix 4). No individual identifier such as names of participants and area of location will be shared. In addition, a confidentiality agreement has been signed with the university and the mobile companies stating that the numbers provided will only be used for the purpose of the trial. All study data and recordings will be destroyed within 5 years of the study according to the recommendation of the UBC and AKU ethics committees.

#### Ethical Considerations

The study protocol and associated study instruments, including consent forms in English and local language, were approved by UBC’s and AKU’s Ethics Review Committee before commencement of any study activities. The study will be conducted in accordance with the Helsinki declaration and established guidelines. All participants will be administered informed consent before participation. Participants have the right to refuse to participate in the study or leave the study at any time; this will not affect any services provided at the health center. Data confidentiality will be maintained at all times. No personal identifiers will be used in any reports or publication of the study.

#### Training and Refresher

Initial training of the study staff related to study protocol, SMS text messages and phone calls will be conducted. This will be followed by refresher training in the middle of the study.

### Limitations or Mitigations

We recognize that not all barriers identified are modifiable and amenable to phone calls and SMS text messages. However, most of them reflect a priority ranking that does not favor child routine vaccination. One major reservation about SMS-based interventions is the recipient’s level of literacy. However, there has been mixed input related to the preference of phones calls as compared with SMS text messages in populations of low literacy and resource-constrained settings [20,27]. Mobile phone SMS text messages in local languages, pictorial messages, and in combination with phone calls can further reduce this gap. In our study, we will send messages in local languages (as per the preference of the participants). Although SMS text messages have a limitation of 160 characters and even fewer if translated in other characters, these limitations might help in making the messages simple and brief, especially for populations with low literacy levels [22,28].

The type of intervention will not be blinded, but all families in the same cluster will receive the same intervention. The primary outcome (vaccine completion) is not likely to be influenced by any knowledge of the intervention arm.

## Result

The baseline survey was conducted from July 2018 to January 2019 where it was found that 97.9% (3797/3877) of care givers either owned or have a sharing access to a working mobile phone. A total of 50 IDIs were conducted before the start of the study, that is, 25 interviews per site, based on which the content of the SMS text messages and automated calls were developed. FGDs with caregivers and stake holders were then conducted to validate the developed content. The RCT enrollment was completed on January 31, 2019, and the participants continued receiving messages till the child was 20-week old. Currently, the study is in its final phase and expecting completion of exit surveys and interviews by June 2019.

## Discussion

This study is the first one of this type to assess the efficacy of different types of SMS text messages and automated calls messages, with or without interactive messages in LMICs. Different types of SMS text messages and automated calls messages will also be personalized based on the identification of families’ possible concerns or challenges regarding RI. Each family will receive one weekly message, and we expect families to talk to each other about the messages within the demographic unit, therefore facilitating circulation of information (this aspect will be assessed through the end-of-study interview). This information will be extremely useful to understand the effect of different types of messages in different contexts.

The qualitative aspect of our study will generate useful information regarding family’s perception of vaccination and the daily life challenges for timely visits to the vaccine clinic. This information will be used for developing more complex interventions that use mobile phone messages and possibly other approaches to overcome some of the barriers. Finally, conducting this study in Pakistan will generate results that will be useful in most LMICs worldwide.

Representatives of public health, Ministry of Health, and community leaders will be part of the study steering committee to ensure transparency, direct communication, and shared decision making as part of on-going knowledge transfer strategy. We will also inform major policy makers, donors, and mobile phone service providers. Study findings will be presented at and published in a scientific journal and national and international scientific and programmatic forums.

## Appendix

Multimedia Appendix 1 Strategy for health messages content.

Multimedia Appendix 2 Example of 1-way and 2-way personalized messages.

Multimedia Appendix 3 Study database.

Multimedia Appendix 4 Study gateway.

Multimedia Appendix 5 Administrative information.

## Abbreviations

AKU: Aga Khan University
EPI: Expanded Programme on Immunization
HDSS: health demographic surveillance systems
IVR: interactive voice recording
LHW: Lady Health Worker
LMIC: low and middle-income country
NB: newborn
RCT: randomized controlled trial
RI: routine immunization
SMS: short message service
UBC: University of British Columbia
VPD: vaccine-preventable disease
