# Supplementary figures and images for: Personalized Text Messages and Automated Calls for Improving Vaccine Coverage Among Children in Pakistan: Protocol for a Community-Based Cluster Randomized Clinical Trial
Source: JMIR Res Protoc. 2019 May 30;8(5):e12851. doi: 10.2196/12851 (PMC6658276; doi:10.2196/12851)

### **Annexure 3**

#### **Strategy for Health Message Content:**

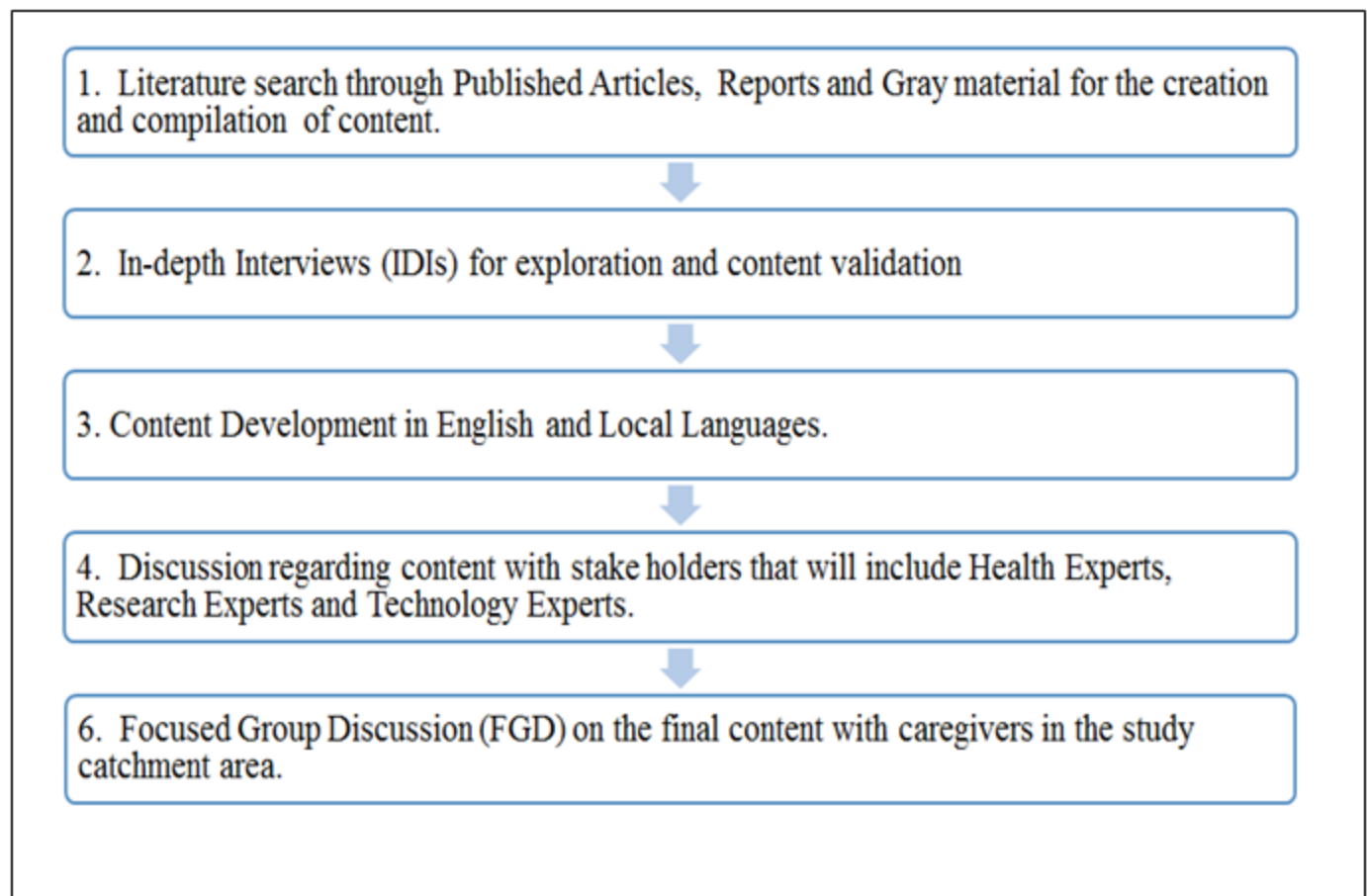

Supplement: Multimedia Appendix 1 [file resprot_v8i5e12851_app1.pdf]
