# Supplementary material for: Personalized Text Messages and Automated Calls for Improving Vaccine Coverage Among Children in Pakistan: Protocol for a Community-Based Cluster Randomized Clinical Trial
Source: JMIR Res Protoc. 2019 May 30;8(5):e12851. doi: 10.2196/12851 (PMC6658276; doi:10.2196/12851)
Supplement: Multimedia Appendix 2 [file resprot_v8i5e12851_app2.pdf]

Annexure 5: Examples of one way and two way SMS messages

	SMS		Automated Calls	
**Type of Messages**	**One Way**	**Two Way**	**One Way**	**Two Way**
**Educational**	***1.AKU (Paigham e Sehat***): Millions of children are protected from dangerous and deadly diseases annually with safety vaccines<br><br>***2. AKU (Paigham e Sehat):*** While visiting the health center keep your vaccination along. Make sure to get the next vaccination information and date written on it.	***AKU (Paigham e Sehat)***: Millions of children are protected from dangerous and deadly diseases annually with safety vaccines. For more information **text** 1 or 2 for more information<br><br>1. Each year 1.4 million children under age of 5 worldwide die due to lack of access to vaccines. For more information type 2<br><br>2. Vaccinations protect those whom you care about. For more information type 1<br><br>2. ***AKU (Paigham e Sehat:)*** While visiting the health center keep your vaccination along. Make sure to get the next vaccination information and date written on it.. For child care ***type*** 1 and for safety type 2<br><br>1. Dont worry while your child is getting vaccinated .If necessary give him a cuddle and breastfeed him. For information on vaccination type 2<br><br>2. Vaccinations will protect your children's health, and the health of your upcoming generations. For information child care type 1	***I am calling from Aga Khan University's Program Paigham e Sehat***. Millions of children are protected from dangerous and deadly diseases annually with safety vaccines	***I am calling from Aga Khan University's Program Paigham e Sehat:*** Millions of children are protected from dangerous and deadly diseases annually with safety vaccines. For more information **press** 1 or 2 for more information<br><br>1. Each year 1.4 million children under age of 5 worldwide die due to lack of access to vaccines. For more information type<br><br>2. Vaccinations protect those whom you care about. For more information type<br><br>2. ***I am calling from Aga Khan University's Program Paigham e Sehat*** :While visiting the health center keep your vaccination along. Make sure to get the next vaccination information and date written on it.. For child care **press** 1 and for safety press 2<br><br>1. Dont worry while your child is getting vaccinated .If necessary give him a cuddle and breastfeed him. For information on vaccination press 2<br><br>2. Vaccinations will protect your children's health, and the health of your upcoming generations. For information child care press 1
**Reminder**	1. (***AKU Paigham e Sehat)***:If you haven't gotten your child's 6th week vaccination, then go to the nearest EPI center/clinic to get that done as soon as possible<br><br>2. AKU(Paigame Sehat) If your child still hasn't received his/her 10th week vaccination, then go to the nearest EPI center to get that done	***AKU (Paigham e Sehat):*** If you haven't gotten your child's 6th week vaccination, then go to the nearest EPI center/clinic to get that done as soon as possible.Did you get your child vaccination. ***Type*** 1 for yes, 2 for no and 3 for do not know<br><br>2. AKU(Paigame Sehat) If your child still hasn't received his/her 10th week vaccination, then go to the nearest EPI center to get that done Did you get your child vaccination. ***Type*** 1 for yes, 2 for no and 3 for do not know	***I am calling from Aga Khan University's Program Paigham e Sehat.*** If you haven't gotten your child's 6th week vaccination, then go to the nearest EPI center/clinic to get that done as soon as possible<br><br>2. I am calling from Aga Khan University's Program Paigham e Sehat :If your child still hasn't received his/her 10th week vaccination, then go to the nearest EPI center to get that done	***I am calling from Aga Khan University's Program Paigham e Sehat:)*** If you haven't gotten your child's 6th week vaccination, then go to the nearest EPI center/clinic to get that done as soon as possible.Did you get your child vaccination. ***Press*** 1 for yes, 2 for no and 3 for do not know<br><br>2. I am calling from Aga Khan University's Program Paigham e Sehat: If your child still hasn't received his/her 10th week vaccination, then go to the nearest EPI center to get that done Did you get your child vaccination. ***Press*** 1 for yes, 2 for no and 3 for do not know
