# Supplementary material for: Personalized Text Messages and Automated Calls for Improving Vaccine Coverage Among Children in Pakistan: Protocol for a Community-Based Cluster Randomized Clinical Trial
Source: JMIR Res Protoc. 2019 May 30;8(5):e12851. doi: 10.2196/12851 (PMC6658276; doi:10.2196/12851)
Supplement: Multimedia Appendix 3 [file resprot_v8i5e12851_app3.pdf]

Annexure 1

Study Data base

A database will also be designed for this study. It will be used during trial phase, which will show the daily status of enrolled NB, total form filled and different variables of our trial questionairre from both sites Karachi and Matiari.

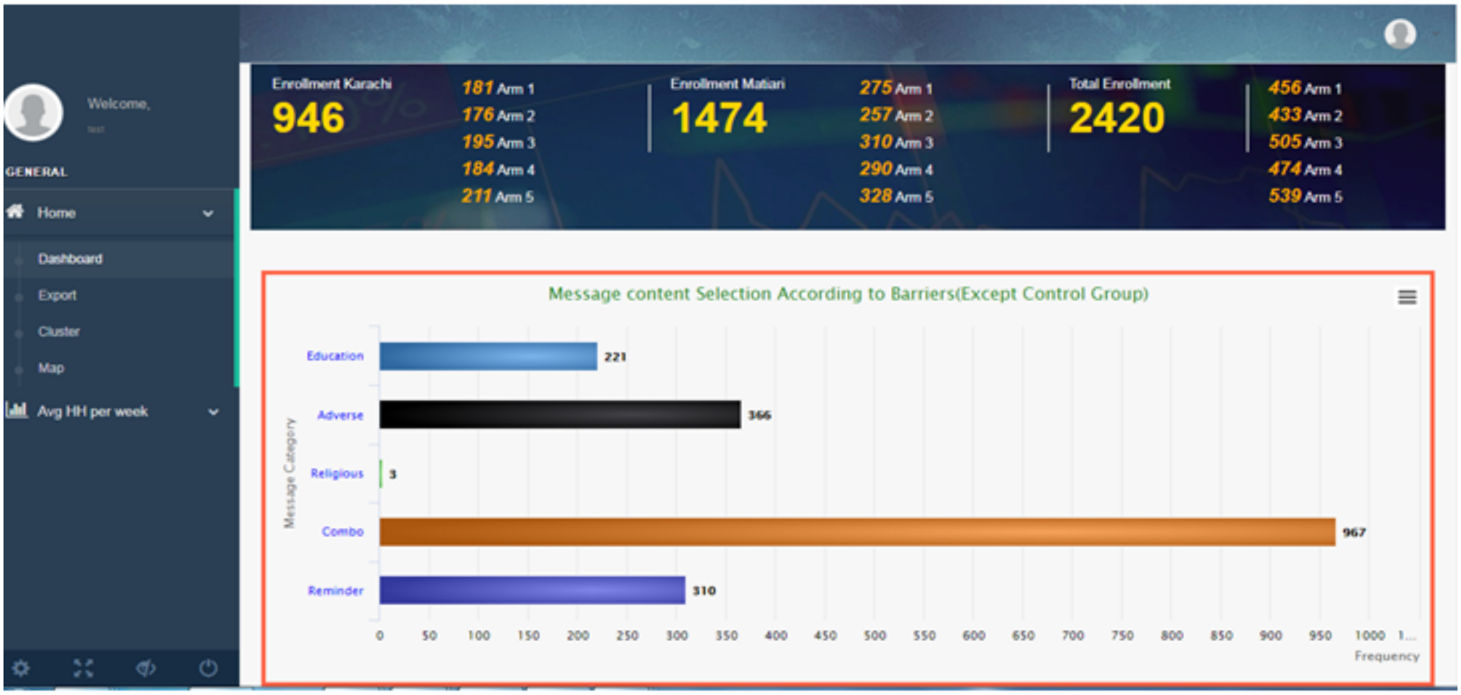

FormFilled

Copy CSV Print

Search:

Consented Data									
Date	BH	IH	IE	AG	KhiTotal	Hala	Saeedabad	Matiari	MatiariTotal
2018-07-18	2	0	1	1	4	3	0	0	3
2018-07-19	3	1	0	0	4	1	0	2	3
2018-07-20	2	4	6	1	13	3	0	3	6
2018-07-21	1	1	2	0	4	0	2	1	3
2018-07-23	4	6	2	3	15	4	0	0	4
2018-07-24	3	4	2	2	11	3	0	0	3
2018-07-26	3	3	2	5	13	6	0	0	6
2018-07-27	1	3	2	2	8	6	0	0	6
2018-07-28	2	1	2	1	6	2	0	4	6
2018-07-30	4	2	1	3	10	6	0	2	8

Showing 1 to 10 of 93 entries

Previous 1 2 3 4 5 ... 10 Next
