# Supplementary material for: Personalized Text Messages and Automated Calls for Improving Vaccine Coverage Among Children in Pakistan: Protocol for a Community-Based Cluster Randomized Clinical Trial
Source: JMIR Res Protoc. 2019 May 30;8(5):e12851. doi: 10.2196/12851 (PMC6658276; doi:10.2196/12851)
Supplement: Multimedia Appendix 4 [file resprot_v8i5e12851_app4.pdf]

Annexure 2

Gate Way

A web portal will designed for this study where we will be able to integrate our SMS and automated call module. This will allow us to send and receive our portal and get the daily counts of sending and receiving the SMS and automated calls

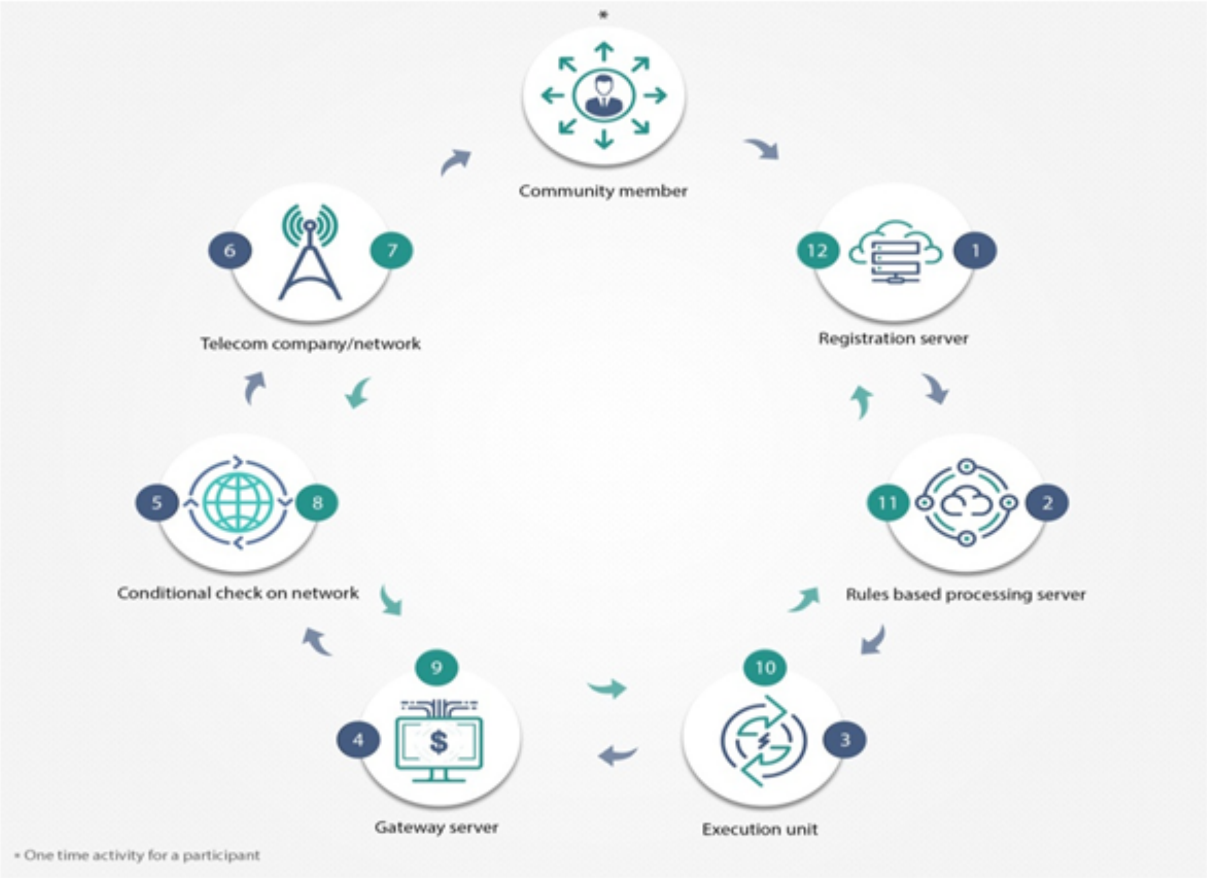

PROCESS	INTERVENTION 1 & 3	INTERVENTION 2 & 4
1	Sends date of birth and phone number	Sends date of birth and phone number
2	Calculates of date of birth	Calculates of date of birth
3	Selects and sends content	Selects and sends content
4	Checks condition on content, service provider and phone number	Checks condition on content, service provider and phone number
5	Sends content and number to respective service provider	Sends content and number to respective service provider
6	Sends content to community member	Sends content to community member
7	No activity	Receives response
8	No activity	Gathers response
9	No activity	Manages with time stamp
10	No activity	Binds data against each participant
11	No activity	Updates status of each slot
12	No activity	Updates status of each participant
