# Supplementary material for: Personalized Text Messages and Automated Calls for Improving Vaccine Coverage Among Children in Pakistan: Protocol for a Community-Based Cluster Randomized Clinical Trial
Source: JMIR Res Protoc. 2019 May 30;8(5):e12851. doi: 10.2196/12851 (PMC6658276; doi:10.2196/12851)
Supplement: Multimedia Appendix 5 [file resprot_v8i5e12851_app5.pdf]

Annexure 4

Administrative Information

Trial Steering Committee (TSC):

The TSC will provide overall supervision of the trial and ensure it is conducted according to acceptable standards. It will monitor the progress of the trial, adherence to protocol, patient safety and consideration of new information. The trial team will consist of the (a)Principal Investigator (PI), who will be responsible for the day-to-day running of all aspects of the trial and for managing the trial budget (b) All co-investigators will part of the committe who will advice on the methodologies (c) Trial coordinators, will follow and report study activities at both sites (d) Staticians, will work and implement data analysis paln (e) Field staff will give input of the field activity and representation of the field sites community.

Data Safety and Monitoring Board (DSMB)

The independent DSMB will have access to all the study data. The role of its members is to monitor these data and make recommendations to the TSC on any ethical or safety issues. Membership will be completely independent of the trial. The DMEC will have 2 independent members including (a) One expert in the field (b) one Independent Expert in clinical. The principal Investigator will be invited to their meetings which will be scheduled for twice a year.
